# HER-2 overexpression differentially alters transforming growth factor-β responses in luminal versus mesenchymal human breast cancer cells

**DOI:** 10.1186/bcr1343

**Published:** 2005-11-08

**Authors:** Cindy A Wilson, Elaina E Cajulis, Jennifer L Green, Taylor M Olsen, Young Ah Chung, Michael A Damore, Judy Dering, Frank J Calzone, Dennis J Slamon

**Affiliations:** 1 Department of Medicine, David Geffen School of Medicine at UCLA, Los Angeles, CA 90095, USA; 2 Amgen Inc., Thousand Oaks, CA 91320, USA; 3 Department of Biology, California Institute of Technology, Pasadena, CA 91125, USA

## Abstract

**Introduction:**

Amplification of the HER-2 receptor tyrosine kinase has been implicated in the pathogenesis and aggressive behavior of approximately 25% of invasive human breast cancers. Clinical and experimental evidence suggest that aberrant HER-2 signaling contributes to tumor initiation and disease progression. Transforming growth factor beta (TGF-β) is the dominant factor opposing growth stimulatory factors and early oncogene activation in many tissues, including the mammary gland. Thus, to better understand the mechanisms by which HER-2 overexpression promotes the early stages of breast cancer, we directly assayed the cellular and molecular effects of TGF-β1 on breast cancer cells in the presence or absence of overexpressed HER-2.

**Methods:**

Cell proliferation assays were used to determine the effect of TGF-β on the growth of breast cancer cells with normal or high level expression of HER-2. Affymetrix microarrays combined with Northern and western blot analysis were used to monitor the transcriptional responses to exogenous TGF-β1 in luminal and mesenchymal-like breast cancer cells. The activity of the core TGF-β signaling pathway was assessed using TGF-β1 binding assays, phospho-specific Smad antibodies, immunofluorescent staining of Smad and Smad DNA binding assays.

**Results:**

We demonstrate that cells engineered to over-express HER-2 are resistant to the anti-proliferative effect of TGF-β1. HER-2 overexpression profoundly diminishes the transcriptional responses induced by TGF-β in the luminal MCF-7 breast cancer cell line and prevents target gene induction by a novel mechanism that does not involve the abrogation of Smad nuclear accumulation, DNA binding or changes in c-myc repression. Conversely, HER-2 overexpression in the context of the mesenchymal MDA-MB-231 breast cell line potentiated the TGF-β induced pro-invasive and pro-metastatic gene signature.

**Conclusion:**

HER-2 overexpression promotes the growth and malignancy of mammary epithelial cells, in part, by conferring resistance to the growth inhibitory effects of TGF-β. In contrast, HER-2 and TGF-β signaling pathways can cooperate to promote especially aggressive disease behavior in the context of a highly invasive breast tumor model.

## Introduction

HER-2 is a member of the type I receptor tyrosine kinase family [1,2], which consists of four closely related family members, HER-2 (*neu*/ErbB2), epidermal growth factor receptor (EGFR; ErbB1), HER-3 (ErbB3) and HER-4 (ErbB4). Unlike the other ErbB family members, HER-2 does not directly bind any ligand with high affinity. Instead, the major role of HER-2 is to serve as a co-receptor in the dimerization and activation of other ErbB receptors [3,4]. Amplification of the *HER-2 *gene is detected in approximately 25% of human breast cancers and this genomic alteration is predictive of poor clinical outcome [5-7]. *HER-2 *amplification results in a 50 to 100-fold increase in the number of surface HER-2 receptors on cancer cells compared to the normal mammary epithelium [8-10]. Aberrant signaling through these receptors is believed to play a direct role in malignant transformation and/or progression. Evidence obtained in model systems supports the premise that progression of *HER-2 *amplified breast cancers is driven by *HER-2 *gene activity. When the level of engineered *HER-2 *expression in tumor cell lines mimics the disease state, important phenotypic changes are observed, including increased growth *in vitro*, decreased anti-estrogen response, increased production of angiogenic factors, as well as increased tumorigenicity and metastatic potential *in vivo*[11-15]. These changes parallel the observed aggressive clinical behavior of human tumors that contain an amplified *HER-2 *gene [5-7].

*HER-2 *gene amplification and oncogenic mutations constitutively activate the HER-2 homodimeric tyrosine kinase [16-18]. Elevated HER-2 activity can reduce the general growth factor dependence of *HER-2 *amplified cells though prolonged stimulation of the Ras/Raf/Mitogen-activated protein kinase (MAPK) pathway [16,17]. It is also increasingly clear that the high cell-surface HER-2 density that accompanies gene amplification alters the normal equilibrium of ErbB dimers in favor of HER-2 containing heterodimers, thus altering ligand dependant signaling mechanisms [19]. The oncogenic potency of heterodimers, EGFR/HER-2 for example, is significantly enhanced compared to EGFR homodimers by several processes that prolong receptor signaling activity [20-22]. The efficient recruitment of the p85 subunit of phosphoinositide 3-kinase by ligand-dependent stimulation of HER-2/HER-3 heterodimers is another important consequence of the shift towards HER-2 containing heterodimers [23]. It is therefore likely that HER-2 induced hypersensitivity to EGF family growth factors may contribute significantly to tumor progression.

*HER-2 *amplification is a relatively early event in the clinical pathogenesis of human breast cancer based on its frequent occurrence in the pre-invasive lesion, ductal carcinoma *in situ *(DCIS) [24-26]. The frequency of *HER-2 *amplification detected in high grade (comedo) DCIS has been reported to be as high as 77% [27]. This evidence suggests that aberrant HER-2 activity plays an important role in tumor initiation as well as in the emergence of aggressive cellular behavior associated with progressive disease. Experimental support for the role of HER-2 in breast cancer initiation comes from transgenic experiments in which wild-type or activated HER-2 expressed in mouse mammary epithelium leads to a high frequency of mammary carcinomas [28-30]. The histopathology of these cancers closely resembles the human malignancy, including the appearance of a DCIS-like lesion followed by invasive disease. It has also been shown that activated HER-2 is uniquely capable of promoting a DCIS-like phenotype in *in vitro *models of mammary acini [31].

In an effort to gain a better understanding of the mechanisms by which elevated HER-2 signaling contributes to tumor initiation, we investigated whether or not HER-2 antagonizes growth inhibitory signals normally present in the breast epithelium. The transforming growth factor beta (TGF-β) signaling pathway is the dominant system opposing the stimulatory effect of growth factors and early oncogene activation in many tissues including the mammary gland [32]. TGF-β exposure markedly suppresses mouse mammary tumor development [32] and reversibly inhibits normal mammary gland growth [33]. It is also well established that TGF-β potently inhibits the growth of normal epithelial cells as well as some breast cancer cell lines in culture [34,35]. Resistance to the anti-proliferative effects of TGF-β appears at an early stage of tumor progression in a number of human malignancies. This occurs in some cancers through mutational inactivation of the TGF-β receptor genes (*TβRII*) or their signaling effectors *SMAD2 *or *SMAD4 *[36-38]. However, the growth inhibitory functions of TGF-β signaling are more commonly subverted by epigenetic changes that reduce receptor expression, prevent the nuclear localization of Smad2 and Smad3 proteins, or functionally inactivate them within a given gene regulatory complex [39].

The current study examines the interaction of the HER-2 and TGF-β signaling pathways in the context of human breast cancer. The primary goal was to examine the potential role for HER-2 overexpression in altering the growth inhibitory activity of TGF-β signaling. The effects of HER-2 overexpression on the TGF-β responses of two estrogen receptor (ER)-positive, luminal breast tumor cell lines, MCF-7 and ZR-75-1, that are experimentally non-invasive were examined. We found that engineered HER-2 overexpression can abrogate TGF-β1 mediated gene responses in both MCF-7 and ZR-75-1 cells and can render the highly sensitive cell line (MCF-7) resistant to the growth inhibitory effects of TGF-β. The functions of TGF-β, however, are not limited to growth inhibition and tumor suppression. TGF-β can also promote invasive cell behavior and metastasis [39] often associated with an epithelial to mesenchymal transition (EMT) [40]. To characterize the interaction of the HER-2 and TGF-β pathways in this context, the effects of HER-2 overexpression on the TGF-β response in the mesenchymal-like breast cancer cell line MDA-MB-231 were examined. In this cellular background, the HER-2 and TGF-β pathways appear to cooperate to promote an especially aggressive phenotype.

## Materials and methods

### Cell lines, antibodies and cytokines

The HER-2 engineered cell lines (MCF-7 H2, ZR-75-1 H2 and MDA-MB-231 H2) were generated by infection with a retroviral vector containing the human, full-length *HER2 *DNA as described previously [13]. Control cell lines (CN) were generated for each cell line by simultaneous infection with the retroviral (pLSXN) vector. Cells were cultured in RPMI 1640 supplemented with 10% v/v fetal bovine serum, 100 U ml^-1 ^penicillin (P) and 100 U ml^-1 ^streptomycin at 37°C in a humidified, 5% CO_2 _atmosphere. Rabbit polyclonal antibodies against p15^INK4B ^and cdk4 (C-22) were purchased from Santa Cruz Biotechnology (Santa Cruz, CA, USA). The mouse monoclonal c-*neu *(Ab-3) antibody was purchased from Oncogene Research Products (San Diego, CA, USA) and those against Smad2 and Smad4 were obtained from Transduction Laboratories (BD, Lexington, KY, USA). Rabbit antibodies against phospho-Smad2, and Smad3 were from Upstate (Lake Placid, NY, USA) and Zymed (San Francisco, CA, USA), respectively. Recombinant human TGF-β1, TGF-β2 and TGF-β3 (R&D Systems, Minneapolis, MN, USA) were diluted in acidified PBS containing 0.1% w/v BSA (diluent control).

### Cell proliferation assays

MCF-7 and ZR-75-1 cells were seeded at 8000 cells/well in 12 well plates and allowed to attach for 12 to 18 h after which treatments were begun (equals day 0). Cells were treated with recombinant TGF-β1 (0.2 to 0.8 ng/ml) and on the indicated days, triplicate wells were harvested by trypsinization and counted using a Coulter counter (Beckman Coulter, Fullerton, CA, USA). MDA MB-231 CN and H2 cells were seeded at 500 cells/well in 96 well plates. After 24 h, increasing concentrations of TGF-β1, TGF-β2, and TGF-β3 (0.01 to 100 ng/ml) were added and the cells were cultured for 6 days. The cells were pulsed with 1 uCi [^3^H] thymidine/well (Perkin Elmer/NEN, Boston, MA, USA) for the final 24 h. Triplicate wells for each data point were harvested by trypsinization and thymidine incorporation was measured using a 96-well TOMTEC harvester (TOMTEC, Hamden, CT, USA).

### RNA preparation and Northern blotting

Total RNA was purified using guanidinium/cesium chloride ultracentrifugation, Trizol reagent (Gibco/Invitrogen, Carlsbad, CA, USA) or RNeasy Midi kits (Qiagen, Valencia, CA, USA). Total RNA (8 or 10 μg) was electrophoresed in 1% glyoxyl gels and transferred to positively charged nylon membranes (Ambion, Austin, TX, USA) using the Turbo Blotter apparatus (Schleicher and Schuell, Keene, NH, USA) and the Northern Max-Gly (Ambion) buffers. The cDNA probes were labeled by random priming using ^32 ^P-dATP (cDNA sequences available upon request). Signals were quantified using a Phosphorimager and ImageQuant software (Molecular Devices, Sunnyvale, CA, USA).

### Microarray analysis

Total RNA was isolated using the RNeasy Mini Kit (Qiagen). The cells were disrupted in approximately 500 ul GITC (guanidine thiocyanate)-containing buffer per 10^6 ^cells. The samples were homogenized by centrifugation (2 minutes at 14 K rpm) through a QIAshredder spin column (Qiagen). The RNA quality was characterized with a RNA 6000 Nano Labchip (Agilent Technologies, Palo Alto, CA, USA). The 28S/18S ribosomal RNA ratios exceeded 1.7 and RNA yields averaged 40 pg per cell. Total RNA was prepared for hybridization following the manufacturer's protocols (Affymetrix, Santa Clara, CA, USA). Fragmented cRNA was hybridized to HGU133A arrays and scanned using a Agilent DNA Microarray Scanner. Expression data were analyzed using Rosetta Resolver 3.0 (Rosetta Informatics, Seattle, WA, USA).

### Cell lysates, western blotting, and Smad2 immunocytochemistry

Semi-confluent cells were harvested with trypsin and lysed in modified RIPA buffer (PBS containing 1.0% v/v Triton X-100, 0.1% v/v SDS, 100 μM Phenylmethylsulfonyl fluoride (PMSF), 10 μg/ml leupeptin and 20 μg/ml aprotinin) at a concentration of 2 to 4 × 10^6 ^cells/ml. Lysates were cleared of insoluble cellular debris by centrifugation, subjected to SDS-PAGE and transferred to PVDF membranes. All buffers, gels and membranes were purchased from Invitrogen. After transfer and blocking in TBS-T (saline buffered with 25 mM Tris-HCl (pH 7.4) containing 0.1% v/v Tween-20) containing 10% w/v nonfat dry milk, membranes were incubated with primary antibodies (diluted in TBS-T + 1% w/v milk) overnight at 4°C. After washing, blots were incubated with anti-mouse or anti-rabbit-HRP (Santa Cruz Biotechnology). Antibody complexes were detected with the ECL chemiluminescent system (Amersham/Pharmacia, Piscataway, NJ, USA). For Smad2 immunohistochemistry, cells were grown for 24 to 48 h on 4 well-chambered slides and treated for 1 h with media containing 2 ng/ml TGF-β1 or diluent control. Cells were then fixed in 4% paraformaldehyde with 0.1% Triton X-100 for 15 minutes, washed in PBS and incubated with the anti-Smad2 antibody diluted 1:500 in blocking buffer (1 × PBS with 2% w/v BSA and 10% v/v normal goat serum) overnight at 4°C. After washing, cells were reacted with a 1:800 dilution of Alexa-488 labeled goat anti-mouse IgG (Molecular Probes, Eugene, OR, USA) for fluorescent visualization. Alternatively for the enzymatic staining, after primary antibody incubation, the cells were incubated with a 1:75 dilution of unlabeled goat anti-mouse IgG followed by a 1:75 dilution of mouse peroxidase anti-peroxidase complex (PAP; Zymed, San Francisco, CA, USA) and antibody complexes were visualized with DAB (3,3' diaminobenzidine) substrate (Sigma, St Louis, MI, USA).

### Ligand binding and DNA binding assays

The Fluorokine kit (R&D Systems) was used to measure binding of fluorescein isothiocyanate (FITC)-labeled recombinant TGF-β1 to the surface of live breast cancer cells. Cells were harvested, filtered to produce single cell suspensions, counted and reacted with labeled TGF-β1 or control protein according to manufacturer's specifications. The Smad DNA binding assay was performed using biotinylated, double-stranded oligonucleotides whose sequence contained the three Smad binding element (SBE) sites and the E-box from the PAI-1 promoter previously described as PE2 [41]. The mutated oligo (PE1^m12,3^) was also synthesized as previously described [41]. Nuclei were isolated from cells treated for 1 h with either diluent control or 2 ng/ml TGF-β1 using the Nuclei EZ lysis buffer as recommended by the manufacturer (Sigma). Nuclear extracts were prepared by resuspending pelleted nuclei in the NER reagent (Pierce, Rockford, IL, USA) followed by vortexing and sonication and finally centrifugation to clear insoluble material. The nuclear extracts were reacted with the wild-type or mutated oligonucleotide for 3 h. DNA-protein complexes were collected using streptavidin-labeled sepharose (Amersham Pharmacia) and after extensive washing, complexes were electrophoresed on SDS-PAGE gels. The presence of Smad2, Smad3 and Smad4 proteins in the complexes was detected by western blotting.

## Results

### Expression profiling reveals that HER-2 overexpression alters components of the TGF-β signaling pathway

We initially performed a genome-wide assessment of the differential gene activity associated with HER-2 overexpression in the MCF-7 breast cancer cell line using cDNA and filter arrays. These transcript profiling data revealed a pattern of changes consistent with a loss of TGF-β signal transduction in MCF-7 cells with elevated levels of HER-2 (Additional file 1). Several TGF-β superfamily ligands and receptors had significantly altered expression in association with HER-2 overexpression. We detected significant expression changes in five TGF-β ligand genes (*TGF-β2, TGF-β3, BMP-3, BMP-5 *and *BMP-7*) and two receptors, the TGF-β type II receptor (*TBRII*) and endoglin. This observation prompted a query of the array data for genes reported to be activated in response to TGF-β. Eight such genes were identified, all of which had significantly lower transcript levels in the MCF-7 H2 cells. A theme of the TGF-β pathway and the presumed function of these genes is the regulation of cell growth and extracellular matrix (ECM) deposition. The products of these eight TGF-β inducible genes include: alpha-1 collagens (type III, V, XVIII); CTGF and CYR61, members of the CCN family of secreted proteins that function as mitoattractants and as regulators of cell migration/adhesion [42]; β-Ig-H3/TGFBI, a secreted protein that has a role in cell-collagen adhesion interactions [43]; TIMP2, an inhibitor of matrix metalloproteinases (such as collagenases) [44,45]; and Endothelin 1 (*ET1*) a secreted protein with vasoconstrictive properties [46]. The consistent suppression of TGF-β activated genes in association with HER-2 overexpression suggests that TGF-β signaling is inhibited in MCF-7 H2 cells. Together these expression changes provided the rationale to further examine the biological consequences and the potential mechanistic interaction of the HER-2 and TGF-β signaling pathways in MCF-7 cells and in additional cell line models.

### Biological effects of TGF-β1 on breast cancer cells with elevated HER-2

**Figure 1:**
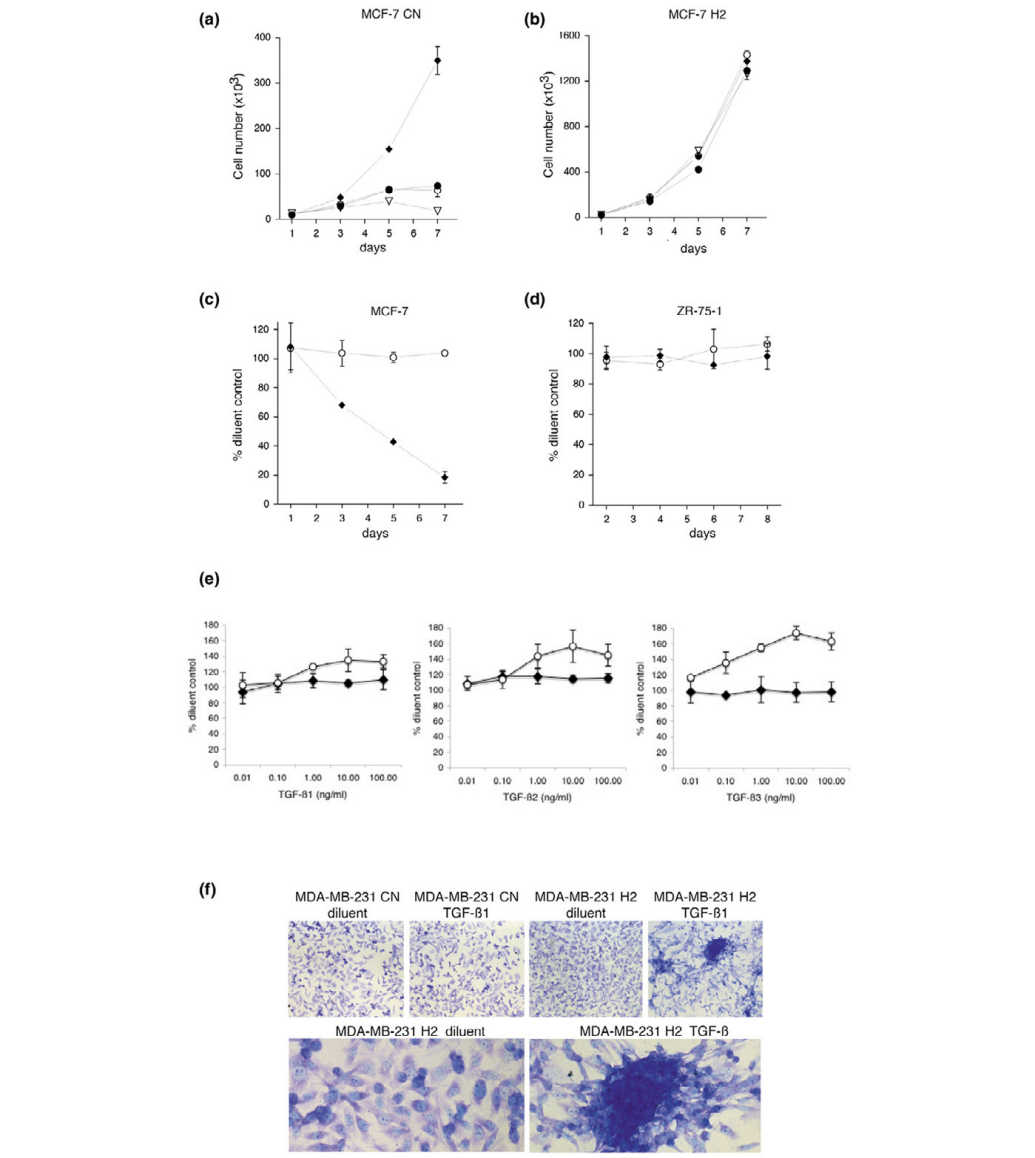
HER-2 overexpression modulates transforming growth factor (TGF)-β responses in a cell context dependent manner. **(a-c) **HER-2 overexpression in MCF-7 breast cancer cells blocks TGF-β mediated growth arrest. CN and H2 cells were treated with diluent control (diamonds) or TGF-β1 (0.2 ng/ml (black circles); 0.4 ng/ml (white circles); or 0.8 ng/ml (triangles)) and counted on the indicated days after treatment. Each point is the average of triplicate wells (± standard deviation for error bars). **(a) **The growth of MCF-7 CN cells is severely inhibited by TGF-β1. **(b) **MCF-7 H2 cells are resistant to growth inhibition by TGF-β1. **(c) **MCF-7 CN (diamonds) versus MCF-7 H2 (white circles) cells treated with 0.2 ng/ml TGF-β1. **(d) **ZR-75-1 cells are resistant to growth inhibition by TGF-β1 without HER-2 overexpression (ZR-75-1/CN (diamonds) versus ZR-75-1/H2 (white circles)) treated with 0.2 ng/ml TGF-β1. **(e) **TGF-β1 stimulates the growth of MDA-MB-231 H2 cells. MDA MB-231 CN (diamonds) and H2 cells (white cirlcles) were grown for 6 days in the presence of TGF-β1, β2 or β3 (0.01 to 100 ng/ml) or a diluent control. Cells were pulsed with [^3^H] thymidine for the final 24 h of assay and thymidine incorporation was measured. The average counts of triplicate wells for each data point are represented as % of diluent control. **(f) **TGF-β induces a 'piling' phenotype in MDA-MB-231 that is dependent on HER-2 overexpression. MDA-MB-231 CN and H2 cells were grown for 5 days in the presence of 10 ng/ml TGF-β1 or diluent control. Cells were stained with crystal violet dye and photographed with a 20× (top four panels) or a 60× (bottom two panels) objective.

Experiments were performed to determine if engineered HER-2 overexpression can alter cellular response to exogenous TGF-β1 in human breast cancer cells. The effect of TGF-β on the growth of previously generated [13] HER-2 overexpressing (H2) and vector control (CN) sub-lines of MCF-7, ZR-75-1 and MDA-MB-231 cells was investigated. A significant difference in TGF-β1 sensitivity was observed in the MCF-7 CN compared to MCF-7 H2 cells (Fig. 1a–c). MCF CN cells grew logarithmically (with a 49-fold increase in cell number) over 7 days of treatment whereas the same cells exposed to TGF-β1 showed only a 2 to 7-fold increase in cell number (Fig. 1a). In contrast, the inhibitory effect of TGF-β1 on MCF-7 H2 cells was minimal, with the number of cells in all treatment groups increasing by 60-fold over 7 days (Fig. 1b). In terms of percent inhibition, the MCF-7 CN cells were 80% growth inhibited at the lowest dose of TGF-β1 whereas the MCF-7 H2 cells were not significantly inhibited (Fig. 1c). The ZR-75-1 CN cells were essentially resistant to growth inhibition by TGF-β1 with or without HER-2 overexpression (Fig. 1d). It has been reported that parental ZR-75-1 cells over-express mdm2, which provides an independent mechanism for acquiring TGF-β1 resistance [47]. The MDA-MB-231 cell line is highly motile and invasive, carries an activated Ki-ras allele and appears phenotypically to have undergone EMT [48-50]. MDA-MB-231 CN cells were resistant to growth inhibition by TGF-β1-3 *in vitro *(Fig. 1e). The MDA-MB-231 H2 cells were not only resistant to growth inhibition by TGF-β1, but were growth stimulated by doses greater than 1 ng/ml of all three TGF-β ligands (Fig. 1e). The MDA-MB-231 cells were also stimulated morphologically to aggregate, forming obvious piles or colonies in culture (Fig. 1f). This effect was not observed in the MDA-MB-231 CN cells, even at relatively high concentrations of TGF-β1 (up to 20 ng/ml). Thus the 'piling' phenotype appears to require both TGF-β1 treatment and HER-2 overexpression.

### Markers of TGF-β pathway activity are reduced in both MCF-7 and ZR-75-1 cells in association with HER-2 overexpression

**Figure 2:**
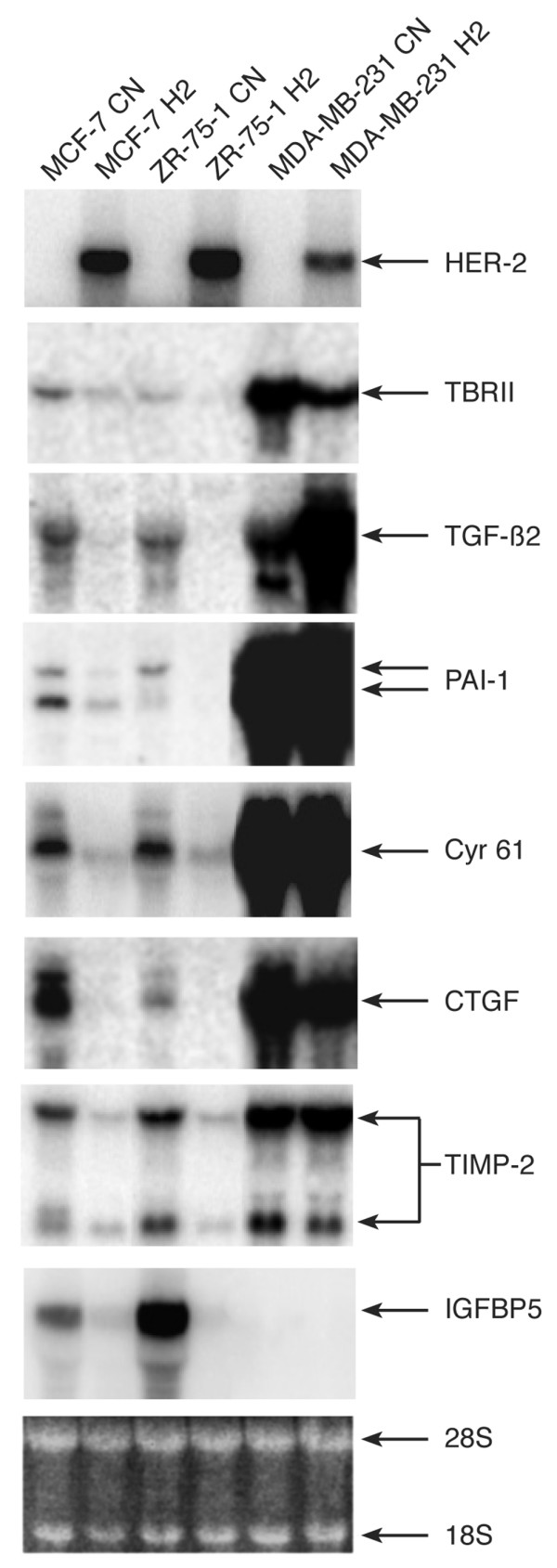
Expression of TGF-β pathway genes in control (CN) and HER-2 overexpressing (H2) breast cancer cells. Northern analysis of HER-2 mRNA and transforming growth factor (TGF)-β markers in MCF-7, ZR-75-1 and MDA-MB-231 cells with (H2) and without (CN) HER-2 overexpression. Message levels were assayed from 10 ug of total RNA. Asterisks indicate consistent and significant down-regulation (> 2-fold as measured by phosphorimager quantification). The image of the 28S and 18S ribosomal RNA bands from the ethidium bromide stained gel is shown as a loading control.

In an attempt to interpret the different biological responses of breast cancer cells to TGF-β treatment, the mRNA expression levels of the *TBRII *and five previously recognized TGF-β response genes identified in our initial expression profiling experiments (Additional file 1), along with the well-characterized TGF-β inducible gene *PAI-1 *[51-53], were evaluated by Northern blotting (Fig. 2). The two ER-positive, luminal cell lines (MCF-7 and ZR-75-1) exhibited very similar TGF-β marker gene expression patterns with low-level *TBRII *and *TGFB2 *expression and low to moderate expression of TGF-β downstream target genes *PAI-1*, *CYR61*, *CTGF*, *TIMP2*, and *IGFBP5*. Conversely, the MDA-MB-231 cells displayed a very different TGF-β marker profile with higher levels (>10-fold) of *TBRII *and *TGFB2*, as well as significantly higher expression of 4/5 of the downstream target genes, *PAI-1*, *CYR61*, *CTGF*, *TIMP2*. These results underscore the differences between cells that have undergone EMT (MDA-MB-231) [49,50] and those that have not (MCF-7 and ZR-75-1) and are consistent with reports demonstrating that TGF-β is a critical mediator of EMT [54-56].

We next assessed the effects of HER-2 overexpression on the TGF-β pathway genes in the matched H2 cell lines. A marked reduction in expression of all the TGF-β pathway markers (*TBRII*, *TGFB2*, *PAI-1*, *CYR61*, *CTGF*, and *TIMP2*) was consistently observed in both MCF-7 and ZR-75-1 cells that overexpressed HER-2 despite their diverse genetic backgrounds and biological properties (Fig. 2). In contrast, expression levels of TGF-β pathway genes were not reduced in association with HER-2 overexpression in the MDA-MB-231 cells. These data suggest that the TGF-β transcriptional program may be generally abrogated in response to HER-2 overexpression in the MCF-7 and ZR-75-1 cells. The high expression of *TGFB2*, *TBRII *and downstream TGF-β target genes observed in the MDA-MB-231 cells suggests that the TGF-β pathway is constitutively activated and, in this context, HER-2 overexpression does not appear to inhibit TGF-β gene responses.

### HER-2 overexpression diminishes the TGF-β1 induced transcriptional program

**Figure 3:**
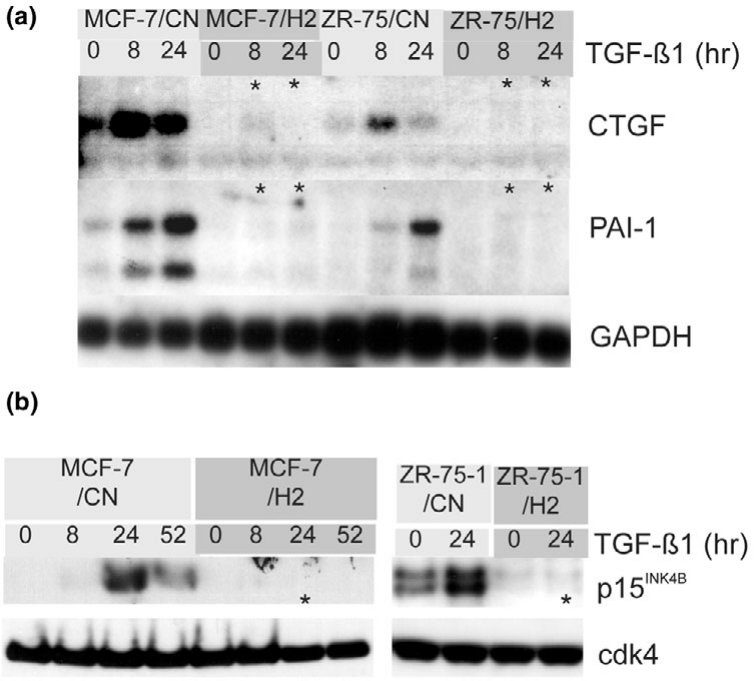
HER-2 overexpression inhibits transforming growth factor (TGF)-β1 target gene induction in the luminal MCF-7 and ZR-75-1 breast cancer cells. **(a) **Northern blot analysis of *CTGF *and *PAI-1 *expression. The membrane contained total RNA from the indicated CN and H2 cells treated with either diluent control (0 h) or 2 ng/ml TGF-β1 (8 or 24 h). **(b) **HER-2 overexpression prevents p15^INK4B ^induction in MCF-7 and ZR-75-1 cells by TGF-β1. Western blot analysis of total cellular extracts prepared after treatment with diluent control (0 h) or 2 ng/ml TGF-β1 (8, 24 or 52 h). Bottom panels are the same membranes re-probed with an antibody to cdk4.

To directly assess whether HER-2 overexpression inhibits TGF-β1 mediated gene induction in the MCF-7 and ZR-75-1 cells, we assayed the expression of three TGF-β target genes, *CTGF*, *PAI-1 *and *p15*^*INK4B *^in response to exogenous, recombinant TGF-β1. These genes contain SBEs and have been extensively utilized to evaluate Smad-dependant TGF-β signaling activity [51,52,57,58]. The MCF-7 CN and ZR-75-1 CN cell lines each showed increased levels of *CTGF *and *PAI-1 *mRNA in response to TGF-β1, with the induction of *CTGF *and *PAI-1 *peaking at 8 and 24 h post TGF-β exposure, respectively (Fig. 3a). Little or no induction of either gene was observed in the same cell lines engineered to overexpress HER-2 (Fig. 3a, asterisks). Moreover, induction of the cdk4 inhibitor p15^INK4B^, a central mediator of TGF-β induced cell cycle arrest [58], was also abrogated by HER-2 overexpression in both MCF-7 and ZR-75-1 cells (Fig. 3b, asterisks).

**Figure 4:**
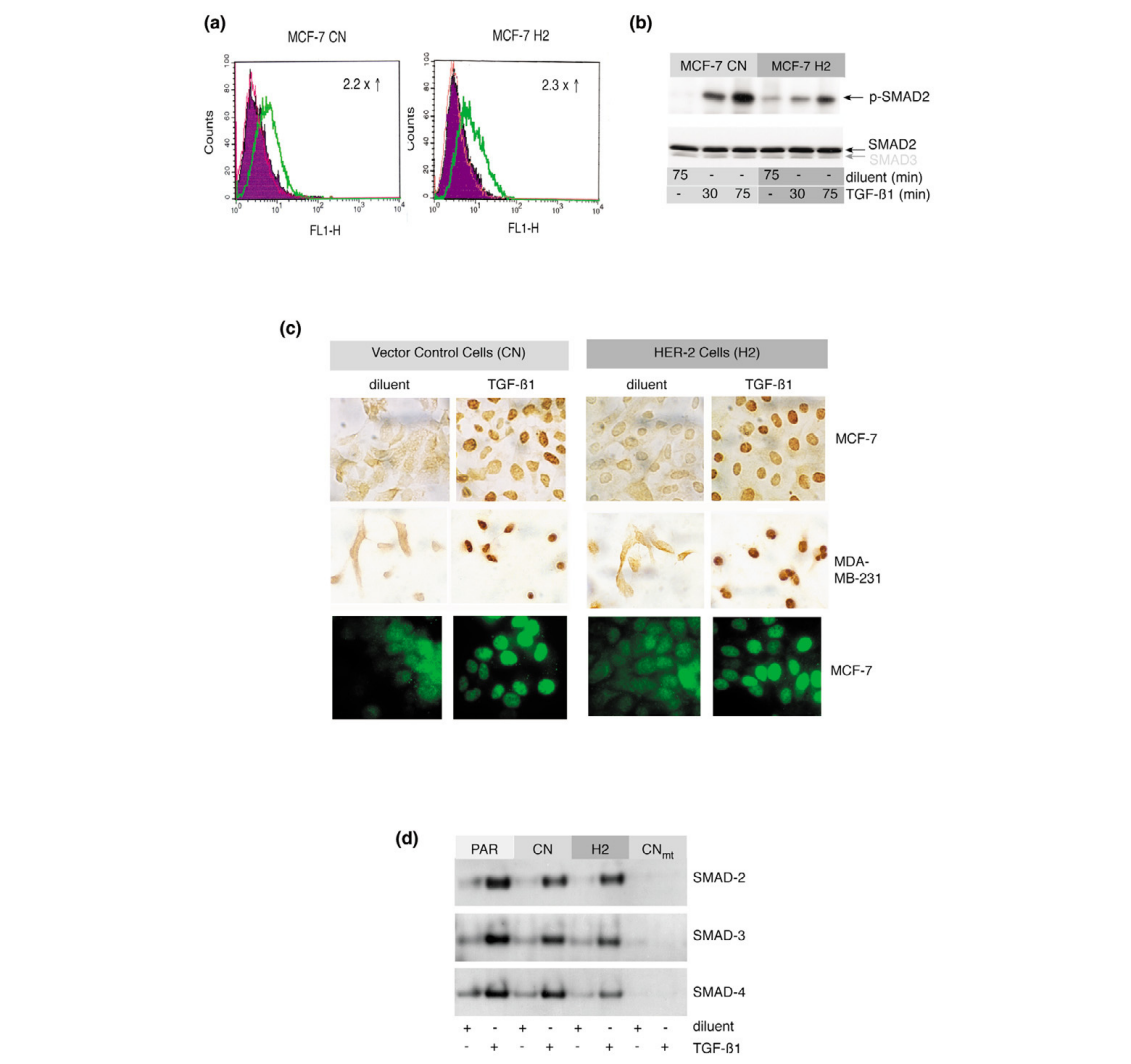
Context specific effects of HER-2 overexpression on the biological responses and transcription program induced by transforming growth factor (TGF)-β in breast cancer cells. **(a) **Phenotypes of the luminal MCF-7 and mesenchymal MDA-MB-231 cells with and without engineered HER-2 overexpression. The epithelial growth pattern of MCF-7 cells is characteristically altered by HER-2 overexpression, which promotes an elongated morphology and increased proliferation rates *in vitro *as well as faster growing tumor xenografts *in vivo *[12,13]. The MDA-MB-231 cells are heterogeneous with the majority of cells, having a spindle shaped morphology. The effect of HER-2 overexpression on the morphological appearance was not dramatic except that significantly more (approximately three times) of the large, flattened round cells were observed. The MDA-MB-231 H2 cells have also been shown to be more metastatic *in vivo *than the MDA-MB-231 CN or parental lines (data not shown). **(b) **Summary of the TGF-β transcriptional program obtained by Affymetrix microarray profiling of cells treated with recombinant TGF-β1. The number of elements significantly affected (p < 0.01 and fold change greater than ± 1.7 using Rosetta Resolver) are graphed. Light and dark red indicate genes upregulated after 6 h and 24 h, respectively, and light and dark green represent genes downregulated after 6 and 24 h, respectively. **(c) **Highlighted genes from the dominant functional gene signatures as determined by the gene ontology information found in Source [105] and GeneCards [106]. Red and green indicate TGF-β stimulated and repressed genes, respectively. ECM, extracellular matrix.

To investigate whether or not HER-2 overexpression affects the global TGF-β gene expression program, microarray experiments were performed. We profiled the expression changes in MCF-7 CN and MCF-7 H2 cells induced by exposure to exogenous, recombinant TGF-β1 for 6 or 24 h (Additional files 2, 3, 4, 5). A 6 h TGF-β exposure resulted in altered expression (>1.5-fold change; p < 0.01) of approximately 0.3% of the total elements represented on the arrays in both the MCF-7 CN and MCF-7 H2 cells (76 and 62 elements, respectively). After 24 h of exposure, this number rose significantly to 352 elements (1.6% of total) in the MCF-7 CN cells but increased only modestly to 81 elements (0.4% of total) in the MCF-7 H2 cells. The 24 h point was included because the PAI-1 and p15^INK4B ^expression data indicated that alterations in key Smad-regulated gene expression can take 24 h to become apparent (Fig. 3). Genes induced or repressed by TGF-β in MCF-7 CN and MCF-7 H2 cells after 24 h were grouped into broad categories (cell cycle, transcription factor, cytoskeleton) by gene ontology (Table 1). The overwhelming majority of the TGF-β response is eliminated in cells that overexpress HER-2 (MCF-7 H2) (Table 1; Fig. 4b). This set of genes includes some early response genes (those present in the 6 h dataset) such as *SMAD3 *(*MADH3*) and the TGF-β inducible early growth response gene (*TIEG*) as well as genes activated later, such as the cytoskeleton associated genes (Paxillin, (*PXN*)). Specifically in terms of TGF-β activated genes, none of the transcription factors, cell cycle or cytoskeleton regulating genes induced by TGF-β in MCF-7 CN were significantly induced in MCF-7 H2 cells (Table 1). The level of *TIEG *expression increased at the 6 h time point in MCF-7 CN cells. It is particularly interesting, however, that this transcriptional activator is not significantly activated in the MCF-7 H2 cells as TIEG has been shown to be a critical mediator of many TGF-β effects and can, by itself, recapitulate the growth inhibitory effects of TGF-β [59-61].

A residual TGF-β gene activation response is observed in the MCF-7 H2 expression profile (Table 1). This includes genes associated with the ECM or cellular adhesion such as collagen V and the Ig superfamily member *IGSF4*, which were similarly induced in the MCF-7 CN and MCF-7 H2 cells. Other genes such as *MSMB *(encodes microseminoprotein beta) were highly induced by TGF-β in MCF-7 CN cells but only moderately induced in the MCF-7 H2 cells.

The most prominent feature in the TGF-β repressed gene set in MCF-7 CN cells is the signature of cell cycle arrest (Table 1). This signature is composed of multiple cyclins (CCNA2, CCNE2, CCNB1 and CCNB2) and S-phase and M-phase specific proteins (CDC2, CDC20, NEK2, and CDC25C) as well as proliferation markers such as Ki67 (*MKI67*), TOPO2A and PCNA. It also includes several mitotic, chromosome segregation and cytokinesis checkpoint and regulating genes (*BUB1*, *CENPA*, *CENPF *and *PRC1*) as well as a large number of genes regulating DNA synthesis, metabolism, and repair (*HELLS*, *LIG1*, *BARD1*, *BRIP1*, *RAD51C*, and *RCF2/4/5*). The majority of changes (31/37; 81%) in the cell cycle arrest profile are absent in the MCF-7 H2 experiments. None of the transcription factors repressed in MCF-7 CN cells, including MXD3 (down-regulated 22-fold), were repressed in the MCF-7 H2 cells. These data are entirely consistent with the TGF-β mediated growth inhibition studies shown above (Fig. 1a).

### HER-2 overexpression abrogates the TGF-β mediated gene response by a novel mechanism

**Figure 5:**
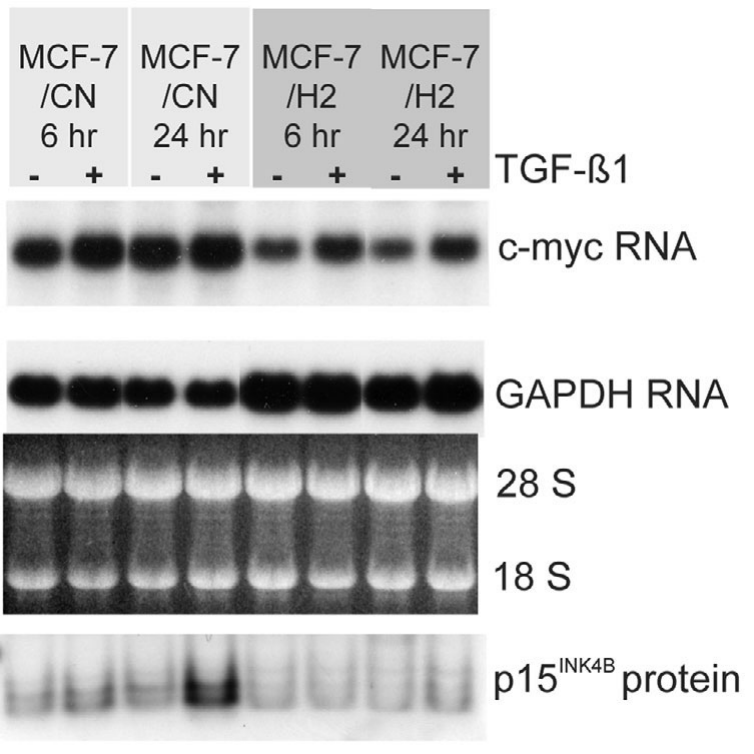
HER-2 overexpression does not alter transforming growth factor (TGF)-β1 induced Smad2 activation or nuclear accumulation or the formation of Smad-Smad-binding element (SBE) complexes. **(a) **TGF-β1 binds equally well to the surface of MCF-7 CN and MCF-7 H2 cells. The binding of fluorescein isothiocyanate (FITC)-labeled recombinant TGF-β1 was monitored by flow cytometry. The red curves show the FL1 values for the untreated control cells, the purple (filled) curves indicate the intensity of cells incubated with an irrelevant FITC-labeled protein and the green curves show the intensity of cells treated with FITC-labeled recombinant TGF-β1. The median shift in FL1 values (fold increase) between the irrelevant control protein and TGF-β1 is indicated. **(b) **TGF-β1 induced phosphorylation of Smad2 is detected in both MCF-7 CN and MCF-7 H2 cells. A phospho-specific Smad2 antibody was used on immunoblots of whole cell lysates from diluent or TGF-β1 treated cells. The blot was re-probed with an antibody against total Smad2, which cross-reacts weakly with Smad3. **(c) **Endogenous Smad2 is concentrated in the nucleus after exposure to TGF-β1 in MCF-7 or MDA-MB-231 cells with or without HER-2 overexpression. Vector control (CN) cells (left panels) or HER-2 overexpressing (H2) cells (right panels) were treated for 1 h with either diluent control or 2 ng/ml TGF-β1, fixed and stained with the anti-Smad2 antibody. The endogenous Smad2 protein was visualized with either a peroxidase/3,3'-Diaminobenzidine (DAB) reaction (top two rows) or an Alexa-488 conjugated secondary antibody (bottom row). **(d) **TGF-β1 stimulated Smad DNA binding is not affected by HER-2 overexpression in MCF-7 cells. Nuclear protein extracts from MCF-7 parental (PAR), MCF-7 CN and MCF-7 H2 cells treated with either diluent or TGF-β1 were reacted with biotin labeled oligonucleotides containing SBEs. Avidin-coupled sepharose beads were used to collect the DNA-protein complexes. The Smad composition of the complexes was analyzed by western blotting with anti-Smad antibodies as indicated. The last two lanes (CN_mt_) contained the same MCF-7 CN nuclear extract as in lanes 3–4, with a mutant oligonucleotide in which one of the SBE sites was mutated (i.e. PE2S^m1 ^[41]).

To evaluate the potential mechanisms by which HER-2 overexpression inhibits TGF-β1 mediated gene activation in luminal breast cancer cells, the status of the core TBRI/TBRII/Smad signal transduction pathway in MCF-7 CN and H2 cells was investigated. Ligand binding was measured using FITC-labeled TGF-β1 and flow cytometric analysis. Evidence of TGF-β1 binding was obtained in both MCF-7 CN and H2 cells as indicated by a 2.2 to 2.3-fold shift in median fluorescence (FL1-H) upon addition of FITC-labeled TGF-β1 to live, single-cell suspensions (Fig. 5a, green curves). The shift in fluorescence was completely blocked by excess unlabeled TGF-β1 or by pre-incubation of the FITC-TGF-β1 with anti-TGF-β1 antibodies (data not shown), indicating that ligand binding was specific. In addition, no shift in fluorescence was observed with a labeled irrelevant protein compared to untreated cells (Fig. 5a). No significant difference in ligand binding was detected in MCF-7 CN compared to MCF-7 H2 cells.

Phosphorylation and nuclear translocation of Smad2 were evaluated next as measures of active receptor status in cells treated with TGF-β1. Phospho-Smad2 was detected after 30 minutes of TGF-β treatment in the MCF-7 CN cells (Fig. 5b). Phospho-Smad2 was also reproducibly detected in the MCF-7 H2 cells, although it was somewhat reduced compared to the CN cells. Endogenous Smad2 was found to translocate to the nucleus equally well in MCF-7 CN and MCF-7 H2 cells after treatment with TGF-β1 for 1 h (Fig. 5c) as measured by immunocytochemistry.

We next investigated whether a defect in TGF-β induced Smad DNA-binding activity could be detected in the MCF-7 H2 cells. Biotinylated oligonucleotides encoding the PE2 element from the PAI-1 promoter [41] were used to examine the extent of association between Smad proteins (Smad2, Smad3 and Smad4) and specific SBEs [62] in response to TGF-β1 stimulation (Fig. 5d). TGF-β1 treatment induced a specific association between Smad2, Smad3 and Smad4 and the wild-type PE2 oligonucleotides whereas no significant association was observed using the control element where the critical first SBE site was mutated (i.e. PE2S^m1^) [41]. The extent of Smad DNA binding was indistinguishable in the MCF-7 parental, CN and H2 cells in this assay. In summary, these data indicate that HER-2 overexpression can abrogate TGF-β1 mediated gene induction without preventing ligand binding, Smad2 nuclear accumulation or Smad DNA binding.

### TGF-β induction of p15^INK4B ^does not depend on c-myc repression in MCF-7 cells

**Figure 6:**
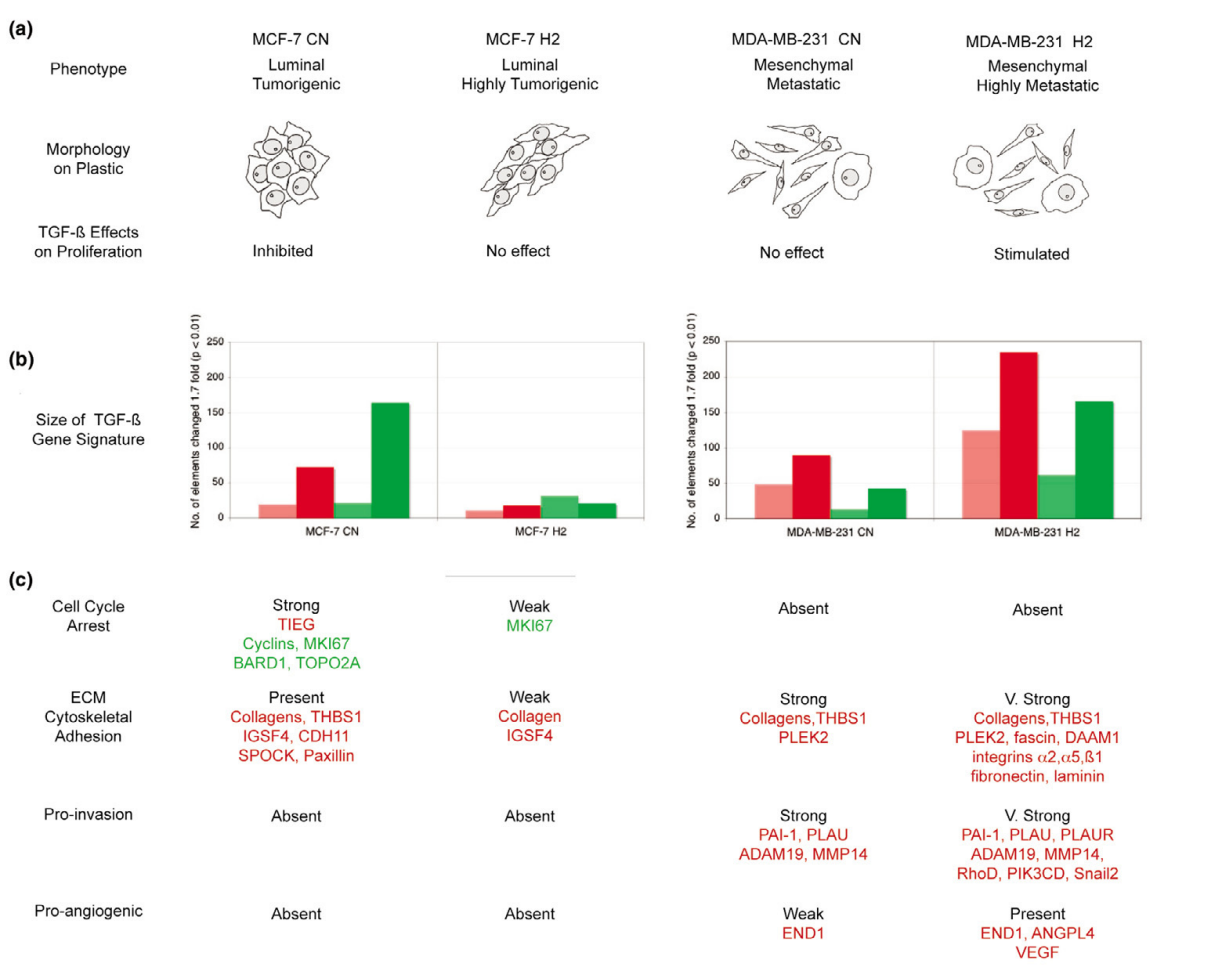
Downregulation of c-myc RNA levels by transforming growth factor (TGF)-β is not observed and is not required for p15^INK4B ^induction in MCF-7 cells. RNA and total protein were extracted from the same flasks of cells treated for either 6 or 24 h with diluent control or 2 ng/ml TGF-β1. The protein samples were probed with an anti-p15^INK4B ^antibody as in Fig. 4b. The Northern blot re-probed with a glyceraldehyde-3-phosphate dehydrogenase (GAPDH) sequence and the image of the 28S and 18S ribosomal RNAs serve as controls.

The repression of c-myc has been shown to be required for the induction of p15^INK4B ^by TGF-β and it has previously been reported that the loss of c-myc repression is central to a TGF-β resistance mechanism in MCF-10A cells transformed by a combination of ras and HER-2 [63,64]. We therefore examined whether or not c-myc expression was different in the MCF-7 CN compared to the MCF-7 H2 cells in response to TGF-β1 treatment. Surprisingly, c-myc mRNA was not repressed by short (6 h) or longer-term (24 h) exposure to TGF-β in the MCF-7 CN or H2 cells (Fig. 6). Instead, a small but reproducible increase (1.3 to 1.4-fold) in c-myc message levels was detected by Northern blot analysis. This same small increase was also confirmed in the transcript ratios detected by the Affymetrix chips. The only difference between the MCF-7 CN and MCF-7 H2 cells with respect to the c-myc message was an overall reduction in the H2 cells (MCF-7 H2 versus CN, ZR-75-1 H2 versus CN and MDA-MB-231 H2 versus CN; Fig. 6 and data not shown). The p15^INK4B ^protein was clearly induced by TGF-β treatment in these same MCF-7 CN cells without repression of c-myc mRNA (Fig. 6). Thus, the transcriptional repression of c-myc does not appear to be critical for the activation of the TGF-β cytostatic gene response or the resulting cell cycle arrest in MCF-7 cells.

### HER-2 overexpression potentiates the TGF-β induced invasion/angiogenic signature in MDA-MB-231 cells

**Figure 7:**
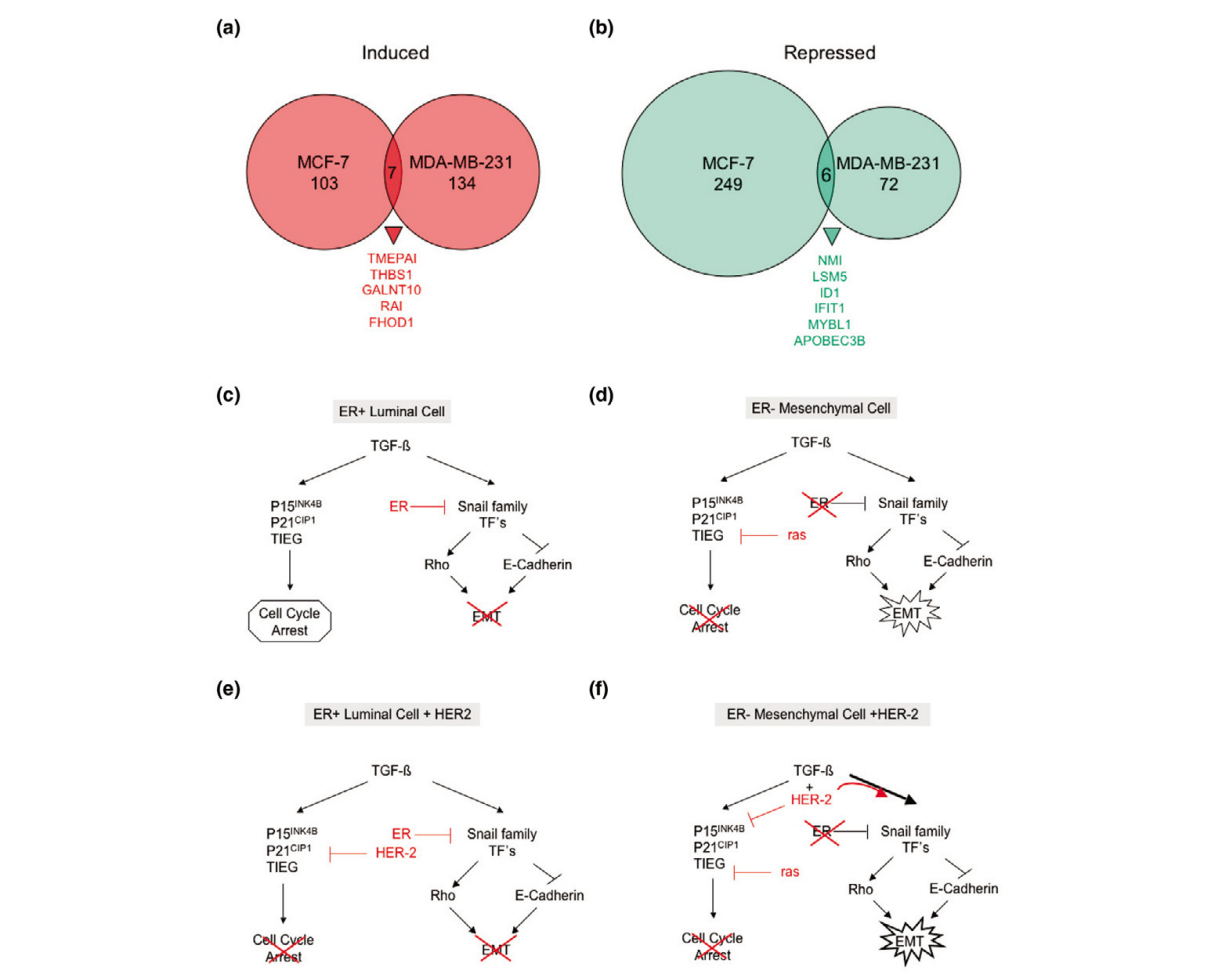
Divergent transforming growth factor (TGF)-β gene activation and biological responses in MCF-7 and MDA-MB-231 cell lines. **(a,b) **The TGF-β induced transcriptional program is almost entirely non-overlapping in the luminal MCF-7 and the mesenchymal MDA-MB-231 breast cancer cell lines. The number of genes **(a) **induced and **(b) **repressed greater than 1.5-fold with a p-value < 0.01 after a 24 h exposure to TGF-β are shown. All of the individual genes commonly affected in both control cell lines (MCF-7 CN and MDA-MB-231 CN) are listed below the diagram. **(c,d) **Model for the differential outcomes of TGF-β stimulation in estrogen receptor (ER) positive luminal breast cells compared to ER-negative mesenchymal cells. **(e,f) **Model for context specific effects of HER-2 overexpression on TGF-β signaling in ER-positive luminal cells versus ER-negative mesenchymal cells. EMT, epithelial to mesenchymal transition.

As we have observed for MCF-7 cells, HER-2 overexpression does not appear to inhibit activation of Smad2 in MDA-MB-231 cells as Smad2 concentrates in the nucleus after TGF-β1 treatment in both MDA-MB-231 CN and MDA-MB-231 H2 cells (Fig. 5c). Thus HER-2 overexpression, oncogenic *ras *[48], or the two combined do not prevent nuclear translocation of Smad2 in response to TGF-β. Nevertheless, we have shown that TGF-β treatment has markedly different biological effects on the luminal MCF-7 cells compared to the mesenchymal-like MDA-MB-231 cells (Fig. 1). In an effort to understand these differential effects, additional microarray profiles were generated for both the MDA-MB-231 CN and H2 cells exposed to exogenous, recombinant TGF-β1 for 6 or 24 h (Additional files 6, 7, 8, 9). A 6 h TGF-β exposure resulted in altered expression (>1.5-fold; p < 0.01) of three times as many elements in the MDA-MB-231 H2 cells (306 elements; 1.4% of the total) as in the MDA-MB-231 CN cells (92 elements; 0.4%). The 24 h exposure affected about twice as many elements as the 6 h time point in both the MDA-MB-231 H2 cells (605 elements; 2.7% of total) and the MDA-MB-231 CN cells (206 elements; 0.9% of total). This overall pattern of gene induction in the MDA-MB-231 cells was very different from that observed in the MCF-7 experiments (Table 2, Figs 4b and 7). There was little overlap (<10%) in the TGF-β signatures from the MCF-7 and MDA-MB-231 cells and HER-2 overexpression in the MDA-MB-231 cell line greatly increased the magnitude and the complexity of the TGF-β gene response rather than abrogating the response as seen in MCF-7 cells (Figs 4b and 7). More genes were induced rather than repressed by TGF-β treatment of MDA-MB-231 cells in contrast with the MCF-7 experiments where the largest subset of differentially regulated genes was found in the MCF-7 CN 24 h repressed group (Fig. 4b). The MCF-7 CN repressed signature was largely composed of a cell cycle arrest profile that was absent in the MDA-MB-231 expression profile.

The majority of the genes differentially regulated by TGF-β exposure in the MDA-MB-231 CN cells were similarly regulated in the MDA-MB-231 H2 cells. A substantial portion of these genes function as components or modulators of the ECM or as regulators of the adhesive properties of cells (Table 2). Included in this list are several genes encoding collagens, metalloproteinases (*ADAM19*, *MMP14*), and secreted factors (*TGFB1*, *LTBB2*, *JAG1*, *WNT5B*) as well as plasminogen regulating genes (*PLAU*, *SERPINE1*/*PAI-1*). The genes outside the MDA-MB-231 (CN and H2) overlap mostly consist of TGF-β gene inductions potentiated by HER-2 overexpression. Many of these TGF-β potentiated genes could be classified as 'pro-malignancy' genes or as genes associated with aggressive, invasive or highly angiogenic tumors. For example, four independent elements representing vascular endothelial growth factor (*VEGF*) were upregulated in the MDA-MB-231 H2 cells, as was the angiopoietin-like molecule ANGPTL4. Other genes associated with invasiveness, cytoskeletal rearrangements, vesicular transport and EMT [65-67], including *PIK3CD*, *FSCN1 *(fascin), *DAAM1*, *SMTN*, *ARHD *(RhoD), *RAB31*, a snail homolog (*SNAI2*) as well as *FN1 *(fibronectin) and *ITGA5 *and *ITGB1 *(fibronectin receptors) were induced in the MDA-MB-231 H2 cells.

## Discussion

The primary objective of the experiments described in this report was to evaluate a potential causal role for HER-2 overexpression in overcoming the growth inhibitory activity of TGF-β signaling in the early stages of breast cancer. The MCF-7 and ZR-75-1 breast cancer cell lines were chosen for this analysis because they display features of 'luminal' differentiation, a property shared by the majority of HER-2 amplified primary breast cancers and cell lines derived from them [68-71]. Luminal cells typically express 'simple' cytokeratins (*KRT8/KRT18*) and generally some detectable level of the ER. The MCF-10A cell line has been previously used as a model to examine the effect of TGF-β and HER-2 in normal human mammary epithelial cells; however, these cells would be better classified as breast 'basal/progenitor' cells because they display *KRT5*, *KRT17*, P-cadherin (*CDH3*) and vimentin (basal markers) as opposed to luminal markers (data not shown) [72]. Although some primary human breast cancers have basal features, these tumors rarely contain the amplified *HER-2 *locus [69,73]. We therefore studied the effects of engineered HER-2 overexpression on TGF-β signaling in the MCF-7 and ZR-75-1 luminal breast cancer cell lines as it is not yet possible to routinely culture normal or immortalized (i.e. non-malignant) luminal mammary epithelial cells.

MCF-7 cells are highly sensitive to activated TGF-β at physiologically relevant concentrations when cultured on plastic, making them a useful model for studying TGF-β mediated growth arrest. The IC_90 _for TGF-β mediated growth inhibition for MCF-7 CN cells was about 10 pM, a dose effectively the same as that defined for this cytokine with the classic mink lung epithelial cell model, Mv1Lu [74]. We show that the potent inhibitory effect of TGF-β1 is essentially eliminated in MCF-7 cells selected for stable overexpression of HER-2. It should be noted that the level of HER-2 receptors in MCF-7 H2 cells is well within the range observed in clinical samples when the gene is amplified [10]. The TGF-β induced gene profiles generated for the MCF-7 CN and MCF-7 H2 cells are entirely consistent with the sensitivity differences to growth inhibition by TGF-β. The majority of the profile detected in the MCF-7 CN cells was not present in the MCF-7 H2 cells, including, most notably, a large set of genes that constitute a clearly recognizable cell cycle arrest signature. This signature is primarily composed of down-regulated genes involved in cell-cycle regulation, chromosomal replication, mitosis, cytokinesis, protein synthesis and general metabolism (Table 1). We have shown by western blot analysis that the cell-cycle arrest response in MCF-7 CN cells includes the induction of the p15^INK4B ^dependent kinase inhibitor that is a direct target of TGF-β induced Smad DNA binding and a central mediator of TGF-β growth arrest [58,63]. The p15^INK4B ^induction is durable for at least 1 to 2 cell-cycle periods, suggesting that the 24 h microarray profiles include primary as well as secondary gene responses.

The induction of well characterized TGF-β target genes, including *p15*^*INK4B*^, *CTGF*, and *PAI-1*, was also found to be abrogated in a second ER-positive, luminal breast cancer cell line, ZR-75-1, when HER-2 is overexpressed (Fig. 3). These cells exhibited a reduction of several key TGF-β pathway markers that was strikingly similar to the pattern observed in MCF-7 H2 cells (Fig. 2). The observation that HER-2 overexpression leads to a similar abrogation of TGF-β signaling in two genetically diverse breast cancer cell lines strengthens the hypothesis that *HER-2* gene amplification contributes to breast cancer progression in part by blocking the potent growth inhibitory signals present in normal breast tissue. This function might be critical to allow a transformed cell previously in contact with the basement membrane to grow unchecked and avoid apoptosis in the center of a breast duct.

Numerous molecular mechanisms for acquired resistance to growth inhibition by TGF-β in epithelial cancers have been proposed. Inactivation of the TGF-β receptor complex, either by deletion or somatic mutation, is important for the genesis of multiple human malignancies [36], although these mutations are uncommon in breast cancer. The downstream signal transduction Smad proteins are also targets of mutational inactivation in some human cancers [37]. Resistance to the anti-proliferation effects of TGF-β in several cell line models, including breast cancer, has been attributed to overexpression of Smad co-repressor proteins [75] such as ski [76,77], sno [78] and evi-1 [79]. Overexpression and/or mutational activation of the oncogenes *c-myc *[80,81] and *ras *[82-84] have been reported to directly render cells resistant to TGF-β. Similarly, amplification and/or overexpression of the *MDM2 *gene have also been associated with TGF-β resistance [47].

It has been previously reported that co-expression of HER-2 and c-Ha-ras can render MCF-10A cells relatively resistant to the growth inhibitory effects of TGF-β [85]. It was proposed that the Smad-dependent repression of c-myc is central to the TGF-β growth arrest program, and that loss of c-myc down-regulation is the critical defect in MCF-10A cells expressing HER-2 and c-Ha-ras [85]. Our results show that induction of p15^INK4B ^expression and the cytostatic effects mediated by TGF-β do not depend on the repression of c-myc mRNA levels in MCF-7 cells (Fig. 4). Therefore, a loss of c-myc repression in MCF-7 H2 cells does not explain the observed TGF-β resistance. MCF-7 cells are not the only example of a cell line potently inhibited by TGF-β without rapid loss of c-myc expression [86,87]. Moreover, it is becoming clear that c-myc independent mechanisms are important for TGF-β growth inhibition, even when rapid transient c-myc down-regulation occurs [88,89].

Our data suggest that defects in HER-2 overexpressing cells that affect TGF-β responses downstream of Smad nuclear accumulation and DNA-binding lead to the generalized loss of growth arrest in luminal breast cancer cells. The elements of the TGF-β pathway required to activate Smad proteins in MCF-7 cells are intact as endogenous Smad proteins translocate to the nucleus and bind to specific SBE elements from the *PAI-1 *promoter equally well in control and HER-2 cells upon treatment with TGF-β1. Thus, the effect of HER-2 overexpression is not analogous to the reported effects of ras on TGF-β signaling where the nuclear translocation of ectopically expressed Smad3 was abrogated in the presence of oncogenic ras [90]. It has been shown that constitutively active raf leads to alterations in TGF-β signaling without affecting Smad nuclear localization [91]. Additionally, the oncogenic *ras *mutations described in the MDA-MB-231 and other cell lines does not prevent the TGF-β stimulated nuclear localization of Smad proteins with or without the addition of high level HER-2 (Fig. 6) [92]. The latter studies demonstrated the nuclear transport of endogenous Smad proteins to the nucleus shortly after TGF-β treatment, even in the absence of Smad4 (as in MDA-MB-468 cells) or in the presence of EGF, activated ras, constitutive raf, or pathologically overexpressed HER-2 (data not shown; Fig. 6).

We used the Affymetrix U133A transcript ratios of the MCF-7 CN compared to the MCF-7 H2 cells to screen for changes in a large number of molecules previously described to participate in, or interact with, the TGF-β signaling pathway [93] (Additional file 10). At least at the level of RNA abundance, we ruled out many potential candidates including the Smad co-repressors ski, sno, SNIP, SIP1 and evi-1. Furthermore, we did not detect expression differences in the inhibitory Smad (I-Smad) proteins in the MCF-7 H2 cells, which rules out one mechanism (transcription up-regulation of *SMAD7*) employed by cytokines such as interferon gamma, tumor necrosis factor-alpha and interleukin-1 to inhibit TGF-β signaling [39]. The most straight forward and promising message differences observed in the MCF-7 CN versus MCF-7 H2 comparisons are the modest but reproducible up-regulation of the protein TGIF [75,94], a homeodomain transcriptional repressor protein thought to recruit histone deacetylases, and decreased expression of two CBP/p300 interacting proteins, PCAF and CITED2. Future work will be needed to validate the possible mechanistic leads that are suggested by these expression data. In addition, the critical defect(s) in the HER-2 overexpressing cells may be the result of post-transcriptional changes that alter specific protein-protein or protein-DNA interactions.

The biological effects and transcriptional program induced by TGF-β in the mesenchymal MDA-MB-231 breast cancer cells is very different from that observed in the luminal breast cancer cells. It is unlikely that the non-overlapping signatures is a result primarily of false positives or random genetic drift as many of the genes induced and repressed in both cell lines (MDA-MB-231 and MCF-7) have been previously described as TGF-β targets in a variety of systems. For example, we found 9/17 genes previously published as TGF-β inducible after 4 h in MDA-MB-231 cells [85] (Table 1) to be similarly induced in the 6 h TGF-β expression profile of MDA-MB-231 CN cells (Additional file 6). Genes encoding ECM components and modifying proteins, as well as genes encoding proteins thought to contribute to motility, invasion and as markers of EMT, were predominantly induced in the MDA-MB-231 cells but not in the MCF-7 cells. Recent data suggest that the snail/slug family of zinc-finger transcriptional repressors are central mediators of EMT, in part by repressing the expression of the tight-junction protein E-cadherin (*CDH1*) and by inducing critical regulators of the cytoskeleton such as RhoD [95-99]. Furthermore, TGF-β can induce snail family proteins in some contexts [100], a link that may help explain the mechanism by which TGF-β contributes to EMT and cancer progression. Recently, it has been shown that the expression of snail is regulated by MTA3, which is in turn regulated by ER signaling [101,102]. The finding that snail expression is blocked by an active ER signaling pathway has critical implications for breast cancer and could potentially explain why the EMT program is not induced by TGF-β in ER-positive breast cancer cells.

The observation that the effects of HER-2 overexpression on TGF-β responses in breast cancer cells is highly context dependent could be explained, for example, by a model in which two major branches of TGF-β responses exist: one that is inhibited by active ER signaling and the other that is inhibited by constitutive, high level ras/MAPK signaling (Fig. 7c,d). This model could also be a framework to explain the composition and size of the TGF-β induced transcriptional response signatures that we observed in each of the four cell lines profiled. Thus, in ER-positive cells without constitutive ras/MAPK signaling (i.e. MCF-7), TGF-β primarily induces a robust cell cycle arrest program (Figs 4 and 7c). HER-2 overexpression without the loss of ER signaling, as is the case in the MCF-7 H2 cells (data not shown), abrogates the TGF-β induced cell cycle arrest program. As the EMT program is still repressed, however, the overall gene expression alterations induced by TGF-β is minimal (Figs 4 and 7e). On the other hand, in an ER-negative cell with a constitutively active ras/MAPK pathway (i.e. MDA-MB-231), TGF-β induces the expression of snail and thereby the expression of an EMT transcriptional program that is almost non-overlapping with the TGF-β signature observed in the MCF-7 cells (Figs 4 and 7d). Finally, when HER-2 is overexpressed in an ER-negative cell (MDA-MB-231 H2), it appears to synergize with the TGF-β pathway to induce an even larger pro-invasion, angiogenesis, and EMT signature (Figs 4 and 7f).

Consistent with our results and this model, HER-2 and the ras/MAPK pathway have been previously reported to synergize with TGF-β signals to promote invasive behavior and metastasis [55,56,103,104]. For example, bitransgenic MMTV-*neu*/MMTV-TGF-β1 mice exhibited higher levels of circulating tumor cells and lung metastasis than the MMTV-*neu *mice and the tumors from the bigenic mice had higher levels of vimentin as well as activated Smad2, Akt, and MAPK [103]. Synergistic effects of HER-2 and TGF-β on the motility of the ER-negative mammary epithelial cell line MCF-10A have also been described [104]. In both of these examples, HER-2 overexpression resulted in an increase in TGF-β mediated Smad activation/activity. Thus, one could hypothesize that increased signaling via the HER-2/ras/MAPK pathway could increase Smad-dependent gene activation and explain the much larger TGF-β signature and biological properties observed in the MDA-MB-231 H2 cells. We have observed evidence of autocrine TGF-β signaling and EMT in a few examples of HER-2 amplified (ER-negative) cancer cell lines, such as SKOV3 and HCC1569 (data not shown). This pro-metastatic activity promoted by HER-2 could explain how the HER-2 amplification event may contribute to clinically late-stage disease and to the particularly aggressive behavior of HER-2 positive tumors [5-7] in addition to its role in breast cancer initiation.

## Conclusion

The gene expression profiles and *in vitro *assays presented in this report demonstrate that the interaction of overexpressed HER-2 and the TGF-β pathway is complex and highly dependent on the cellular background. In luminal breast cancer cells, HER-2 overexpression can block TGF-β mediated cell cycle arrest by a previously unreported mechanism that does not involve the abrogation of Smad nuclear accumulation, DNA binding or changes in c-myc repression. Conversely, in the post-EMT context, HER-2 and TGF-β can cooperate to increase the malignant potential of breast cancer cells. These latter, seemingly synergistic effects of elevated HER-2 and TGF-β signaling could provide a rationale for using combined biological therapies that target these two pathways.

## Abbreviations

BSA = bovine serum albumin; DCIS = ductal carcinoma *in situ*; ECM = extracellular matrix; EGFR = epidermal growth factor receptor; EMT = epithelial to mesenchymal transition; ER = estrogen receptor; FITC = fluorescein isothiocyanate; MAPK = mitogen-activated protein kinase; PBS = phosphate-buffered saline; SBE = Smad-binding element; TGF-β = transforming growth factor beta.

## Competing interests

DJS, CAW and FIC have a related patent pending.

## Authors' contributions

CAW, FJC and DJS conceived of the study and participated in its design. CAW and FJC coordinated the experiments and drafted the manuscript. EEC isolated RNAs and performed Northern blotting analysis, JLG carried out the assays to determine the activity and location of the Smad proteins, and TMO and YAC carried out the cell proliferation assays. MAD carried out the Affymetrix microarray experiments and JD helped with the analysis of the microarray expression data. All authors read and approved the final manuscript.

## Supplementary Material

Additional File 1 Transcript profiling of HER-2 overexpressing MCF-7 breast cancer cells reveals TGF-β pathway alterations. Schematic of results obtained using cDNA microarrays (Synteni/Incyte) and filter blots (Atlas Cancer Blot). Changes in 274 microarray elements representing 189 individual genes were detected. The remaining changes (216 elements) occurred in EST sequences or cDNAs with no published information. Sixteen genes from the cDNA arrays and ten from the filter arrays (18 total as 8 were overlapping) related to the TGF-β pathway are listed by name and GenBank number. Fold differences in green signify a higher signal in MCF-7/CN cells and red numbers signify a higher signal in the MCF-7/H2 cells. Asterisks after the fold differences denote differential hybridization of multiple elements followed by the number of elements that were averaged to give the final fold change. Each gene listed in black was included in this TGF-β pathway signature based on published observations. Click here for file

Additional File 2 A table (Excel file) listing the effects of 6 h TGF-β exposure on MCF-7 CN cells. It includes 76 elements (0.3% of the total 22,283 elements queried) up- or down-regulated greater than 1.5-fold with a p-value < 0.01. Click here for file

Additional File 3 A table (Excel file) listing the effects of 24 h TGF-β exposure on MCF-7 CN cells. It includes 352 elements (1.6% of total) up- or down-regulated greater than 1.5-fold with a p-value < 0.01. Click here for file

Additional File 4 A table (Excel file) listing the effects of 6 h TGF-β exposure on MCF-7 H2 cells. It includes 62 elements (0.3% of total) up- or down-regulated greater than 1.5-fold with a p-value < 0.01. Click here for file

Additional File 5 A table (Excel file) listing the effects of 24 h TGF-β exposure on MCF-7 H2 cells. It includes 81 elements (0.4% of total) up- or down-regulated greater than 1.5-fold with a p-value < 0.01. Click here for file

Additional File 6 A table (Excel file) listing the effects of 6 h TGF-β exposure on MDA-MB-231 CN cells. It includes 92 elements (0.4% of total) up- or down-regulated greater than 1.5-fold with a p-value < 0.01. This table also contains the overlapping TGF-β induced signature obtained in a similar, previously published experiment [85]. Click here for file

Additional File 7 A table (Excel file) listing the effects of 24 h TGF-β exposure on MDA-MB-231 CN cells. It includes 206 elements (0.9% of total) up- or down-regulated greater than 1.5-fold with a p-value < 0.01. Click here for file

Additional File 8 A table (Excel file) listing the effects of 6 h TGF-β exposure on MDA-MB-231 H2 cells. It includes 306 elements (1.4% of total) up- or down-regulated greater than 1.5-fold with a p-value < 0.01. Click here for file

Additional File 9 A table (Excel file) listing the effects of 24 h TGF-β exposure on MDA-MB-231 H2 cells. It includes 605 elements (2.7% of total) up- or down-regulated greater than 1.5-fold with a p-value < 0.01. Click here for file

Additional File 10 Table (Excel file) of the expression differences in MCF-7 CN versus MCF-7 H2 cells of genes in, or that modulate, the TGF-β signaling pathway. Genes listed in red are higher in MCF-7 H2 relative to MCF-7 CN and those listed in green are lower in MCF-7 H2 relative to MCF-7 CN. The first four columns are from one experiment and the second four columns are from an independent experiment using different treated flasks of cells. Click here for file

## Figures and Tables

**Table 1:** Dominant transforming growth factor-β gene signatures in the luminal MCF-7 CN and MCF-7 H2 cell lines

Sequence name^a^	Sequence description^b^	Fold Δ MCF-7 CN^c^	Fold Δ MCF-7 H2^d^	Gene ontology^e^
TGF-β induced genes				
*BCL3*	B-cell chronic lymphocytic Leukemia (CLL)/lymphoma 3	1.70		Cell cycle
*BACE*	Beta-site amyloid precursor protein (APP)-cleaving enzyme	2.86		ECM/adhesion
*CD59*	CD59 antigen p18-20	1.74 (3)		ECM/adhesion
*CDH11*	Cadherin 11, type 2, OB-cadherin (osteoblast)	1.94		ECM/adhesion
*COL18A1*	Collagen, type XVIII, alpha 1	1.91		ECM/adhesion
*COL5A1*	Collagen, type V, alpha 1	5.01 (2)	4.22 (2)	ECM/adhesion
*IGSF4*	Immunoglobulin superfamily, member 4	1.94	1.81	ECM/adhesion
*SCARB1*	Scavenger receptor class B, member 1	1.77		ECM/adhesion
*SPOCK*	Sparc/osteonectin (testican)	8.23		ECM/adhesion
*THBS1*	Thrombospondin 1	2.31 (3)		ECM/adhesion
*BMP7*	Bone morphogenetic protein 7 (osteogenic protein 1)	2.05		Secreted factor
*IGFBP5*	Insulin-like growth factor binding protein 5	4.56		Secreted factor
*MSMB*	microseminoprotein, beta-	11.19 (2)	3.65 (2)	Secreted factor
*CBFA2T3*	Core-binding factor, runt domain, alpha subunit 2	2.06		Transcription factor
*DZIP3*	Zinc finger DAZ interacting protein 3	1.93		Transcription factor
*ELK3*	Sapiens cDNA: FLJ22425 fis, clone HRC08686	1.90		Transcription factor
*FOXO1A*	Forkhead box O1A (rhabdomyosarcoma)	3.45 (2)		Transcription factor
*MADH3*	MAD, mothers against decapentaplegic homolog 3	4.77		Transcription factor
*NRBP*	Nuclear receptor binding protein	2.12		Transcription factor
*PLU-1*	Putative DNA/chromatin binding motif	1.75 (2)		Transcription factor
*SOLH*	Small optic lobes homolog (*Drosophila*)	1.86		Transcription factor
*TGIF*	Transforming growth factor beta (TGFB)-induced factor (TALE family homeobox)	1.70		Transcription factor
*TIEG*	Transforming growth factor beta (TGFB) inducible early growth response	1.77		Transcription factor
TGF-β repressed genes				
*BIRC5*	Baculoviral inhibitor of apoptosis (IAP) repeat-containing 5 (survivin)	-3.04		Apoptosis
*CCNA2*	Cyclin A2	-2.18		Cell cycle
*CCNB1*	Cyclin B1	-1.99		Cell cycle
*CCNB2*	Cyclin B2	-1.94		Cell cycle
*CCNE2*	Cyclin E2	-2.03		Cell cycle
*CDC2*	Cell division cycle 2, G1 to S and G2 to M	-2.62 (2)	-1.77 (2)	Cell cycle
*CDC20*	CDC20 cell division cycle 20 homolog (*S. cerevisiae*)	-2.10		Cell cycle
*CDC25C*	Cell division cycle 25C	-2.02		Cell cycle
*CDKN2C*	Cyclin-dependent kinase inhibitor 2C (p18, inhibits CDK4)	-2.59		Cell cycle
*CDKN3*	Cyclin-dependent kinase inhibitor 3	-2.66		Cell cycle
*CKS1B*	CDC28 protein kinase regulatory subunit 1B	-1.96		Cell cycle
*CKS2*	CDC28 protein kinase regulatory subunit 2	-1.70		Cell cycle
*MKI67*	Antigen identified by monoclonal antibody Ki-67	-2.26 (2)	-2.84	Cell cycle
*MPHOSPH9*	M-phase phosphoprotein 9	-1.86		Cell cycle
*NEK2*	NIMA (never in mitosis gene a)-related kinase 2	-2.93		Cell cycle
*ASPM*	asp (abnormal spindle)-like, microcephaly associated	-2.48		Chromosome reg.
*BUB1*	BUB1 budding uninhibited by benzimidazoles 1 homolog	-2.08	-1.72	Chromosome reg.
*BUB1B*	BUB1 budding uninhibited by benzimidazoles 1 homolog β	-1.78		Chromosome reg.
*CENPA*	Centromere protein A, 17 kDa	-2.53		Chromosome reg.
*CENPE*	Centromere protein E, 312kDa	-9.38	-4.87	Chromosome reg.
*CENPF*	Centromere protein F, 350/400 kDa (mitosin)	-2.42		Chromosome reg.
*CNAP1*	Chromosome condensation-related Structural maintenance of chromosomes (SMC)-associated protein 1	-1.99		Chromosome reg.
*ESPL1*	Extra spindle poles like 1 (*S. cerevisiae*)	-2.62		Chromosome reg.
*HCAP-G*	Chromosome condensation protein G	-2.29 (2)	-1.90	Chromosome reg.
*HEC*	Highly expressed in cancer, rich in leucine heptad repeats	-4.35	-2.32	Chromosome reg.
*PRC1*	Protein regulator of cytokinesis 1	-2.23		Chromosome reg.
*SMC2L1*	SMC2 structural maintenance of chromosomes 2-like 1	-3.27	-1.70	Chromosome reg.
*ZWINT*	ZW10 interactor	-1.75		Chromosome reg.
*BARD1*	BRCA1 associated RING domain 1	-1.92		DNA metabolism
*BRIP1*	BRCA1 interacting protein C-terminal helicase 1	-1.71		DNA metabolism
*CDT1*	DNA replication factor	-1.70		DNA metabolism
*PIR51*	RAD51-interacting protein	-2.17		DNA metabolism
*POLD1*	Polymerase (DNA directed), delta 1, catalytic subunit	-2.68		DNA metabolism
*POLE2*	Polymerase (DNA directed), epsilon 2 (p59 subunit)	-1.78		DNA metabolism
*PRIM1*	Primase, polypeptide 1, 49 kDa	-1.76		DNA metabolism
*RAD51C*	RAD51 homolog C (*S. cerevisiae*)	-1.72		DNA metabolism
*RFC4*	Replication factor C (activator 1) 4, 37 kDa	-1.72		DNA metabolism
*TOP2A*	Topoisomerase (DNA) II alpha 170 kDa	-2.68 (2)		DNA metabolism
*ID1*	Inhibitor of DNA binding 1, dominant negative HLH protein	-1.74		Transcription factor
*MXD3*	MAX dimerization protein 3	-22.10		Transcription factor
*MYBL1*	v-myb myeloblastosis viral oncogene homolog-like 1	-2.09		Transcription factor
*MYBL2*	v-myb myeloblastosis viral oncogene homolog-like 2	-2.07		Transcription factor

^a,b^The primary sequence names and descriptions for the differentially regulated genes (defined as greater or equal to 1.7-fold changed with a p-value of < 0.01) were extracted using Rosetta Resolver. Genes from the dominant ontology classes are shown and thus genes with unknown function or those not in well represented ontology classes are not listed. The complete set of differentially expressed genes can be found in Additional files 2, 3, 4, 5. ^c^Fold change in expression of the induced or repressed genes in the MCF-7 CN cell line after a 24 h exposure to 2 ng/ml recombinant transforming growth factor (TGF)-β1 compared to the diluent treated controls. The number in parentheses after the fold change indicates the number of affected elements that were averaged to calculate the fold difference. ^d^Fold change in expression of the induced or repressed genes in the MCF-7 H2 cell line after a 24 h exposure to 2 ng/ml recombinant TGF-β1 compared to the diluent treated controls. The number in parentheses to the right of the fold change indicates the number of affected elements that were averaged to calculate the fold difference. ^e^The gene ontology annotation was curated from the Summary Function and GO fields downloaded from SOURCE [105].

**Table 2:** Dominant gene signatures induced by transforming growth factor-β1 in the mesenchymal MDA-MB-231 CN and MDA-MB-231 H2 cell lines

Sequence name^a^	Sequence description^b^	Fold Δ MB-231 CN^c^	Fold Δ MB-231 H2^d^	Gene ontology^e^
*ANGPTL4*	Angiopoietin-like 4		3.34	Angiogenesis
*EDN1*	Endothelin 1 (ET-1)	2.87	2.45	Angiogenesis
*VEGF*	Vascular endothelial growth factor		2.16 (4)	Angiogenesis
*DAAM1*	Dishevelled associated activator of morphogenesis 1		3.58	Cytoskeleton regulation
*FSCN1*	Fascin homolog 1, actin-bundling protein		2.71	Cytoskeleton regulation
*KRT7*	Keratin 7		1.71	Cytoskeleton regulation
*KRTHB1*	Keratin, hair, basic, 1		1.70	Cytoskeleton regulation
*MYO10*	Myosin X	2.13	2.16	Cytoskeleton regulation
*NEDD9*	Neural precursor cell, developmentally down-regulated 9		2.56	Cytoskeleton regulation
*PLEK2*	Pleckstrin 2	4.62	3.80	Cytoskeleton regulation
*PODXL*	Podocalyxin-like	3.07	1.92	Cytoskeleton regulation
*PDLIM4*	LIM domain protein, Reversion-induced LIM protein (RIL)		1.95	Cytoskeleton regulation
*SMTN*	Smoothelin		2.02	Cytoskeleton regulation
*SPAG4*	Sperm associated antigen 4		2.60	Cytoskeleton regulation
*TAGLN*	Transgelin	2.21	3.35	Cytoskeleton regulation
*TPM1*	Tropomyosin 1 (alpha)	1.90 (3)	2.42 (3)	Cytoskeleton regulation
*TUBA3*	Tubulin, alpha 3		2.04	Cytoskeleton regulation
*COL1A1*	Collagen, type I, alpha 1	10.10	18.47 (2)	ECM/adhesion
*COL4A1*	Collagen, type IV, alpha 1	5.08 (2)	3.94 (2)	ECM/adhesion
*COL4A2*	Collagen, type IV, alpha 2	3.10 (2)	2.40 (2)	ECM/adhesion
*COL5A1*	Collagen, type V, alpha 1		3.71 (3)	ECM/adhesion
*COL6A3*	Collagen, type VI, alpha 3		20.61	ECM/adhesion
*COL7A1*	Collagen, type VII, alpha 1		3.74 (2)	ECM/adhesion
*FN1*	Fibronectin 1		2.22 (4)	ECM/adhesion
*ITGA2*	Integrin, alpha 2 (CD49B, alpha 2, VLA-2 receptor)		1.85	ECM/adhesion
*ITGA5*	Integrin, alpha 5 (fibronectin receptor)		2.58	ECM/adhesion
*ITGB1*	Integrin, beta 1 (fibronectin, receptor, beta)		3.13	ECM/adhesion
*LAMC2*	Laminin, gamma 2		7.84 (2)	ECM/adhesion
*MFAP2*	Microfibrillar-associated protein 2		2.15	ECM/adhesion
*SDC1*	Syndecan 1		2.25 (2)	ECM/adhesion
*THBS1*	Thrombospondin 1	5.15 (4)	3.03 (4)	ECM/adhesion
*CLDN4*	Claudin 4		1.78	ECM/adhesion
*ADAM19*	A disintegrin and metalloproteinase domain 19	2.42	3.12	Protease and inhibitors
*FURIN*	Furin (paired basic amino acid cleaving enzyme)	2.57	2.56	Protease and inhibitors
*MMP14*	Matrix metalloproteinase 14 (membrane-inserted)	2.19	1.85 (2)	Protease and inhibitors
*PRSS3*	Protease, serine, 3 (mesotrypsin)		3.19 (2)	Protease and inhibitors
*PLAU*	Plasminogen activator, urokinase	2.02 (2)	2.76 (2)	Protease and inhibitors/ECM
*PLAUR*	Plasminogen activator, urokinase receptor		1.70	Protease and inhibitors/ECM
*SERPINE1*	PAI-1 plasminogen activator inhibitor type 1	5.80 (2)	3.74 (3)	Protease and inhibitors/ECM
*BMP1*	Bone morphogenetic protein 1		2.29	Secreted factor
*FSTL3*	Follistatin-like 3 (secreted glycoprotein)	2.31	3.00	Secreted factor
*IL11*	Interleukin 11	2.78	2.13 (2)	Secreted factor
*JAG1*	Jagged 1 (Alagille syndrome)	1.98	1.99	Secreted factor
*LTBP2*	Latent transforming growth factor-β binding protein 2	2.44	3.12	Secreted factor
*LTBP3*	Latent transforming growth factor-β binding protein 3		3.26	Secreted factor
*STC1*	Stanniocalcin 1	2.99 (2)	2.01 (3)	Secreted factor
*TGFB1*	Transforming growth factor, beta 1	5.77	3.98 (2)	Secreted factor
*WNT5B*	Wingless-type MMTV integration site family, 5B	1.80	5.82	Secreted factor
*CGB*	Chorionic gonadotropin, beta polypeptide	18.25	45.95	Secreted factor
*ARHB*	RhoB	2.83	1.91	Signal transduction
*ARHD*	ras homolog gene family, member D		9.26	Signal transduction
*CSF1R*	Colony stimulating factor 1 receptor, (v-fms) oncogene		7.16	Signal transduction
*EPHB2*	EphB2	5.09	6.30 (4)	Signal transduction
*MADH7*	MAD, mothers against decapentaplegic homolog 7	2.16	5.79	Signal transduction
*MAPK13*	Mitogen-activated protein kinase 13		2.28	Signal transduction
*MAPK8IP3*	Mitogen-activated protein kinase 8 interact. protein 3		2.02	Signal transduction
*PIK3CD*	Phosphoinositide-3-kinase, catalytic, delta polypeptide		2.46	Signal transduction
*RAB31*	RAB31, member RAS oncogene family		1.82	Signal transduction
*RALB*	v-ral simian leukemia viral oncogene homolog B		1.93	Signal transduction
*SGNE1*	Secretory granule, neuroendocrine protein 1		10.38	Signal transduction
*SPEC1*	Small protein effector 1 of Cdc42	1.92	1.97	Signal transduction
*TMEPAI*	Transmembrane, prostate androgen induced RNA	7.85	9.08	Signal transduction
*ABL1*	v-abl Abelson murine leukemia viral oncogene 1		1.95	Signal transduction
*NET1*	Neuroepithelial cell transforming gene 1		2.25 (2)	Signal transduction
*PINK1*	phosphatase and tensin homolog (PTEN) induced putative kinase 1		1.98 (2)	Signal transduction
*PORCN*	Likely ortholog of mouse porcupine homolog, PPN		2.49	Signal transduction
*PTPRK*	Protein tyrosine phosphatase, receptor type, K	1.85	2.29	Signal transduction
*JUN*	v-jun sarcoma virus 17 oncogene homolog (avian)		1.98 (2)	Transcription factor
*JUNB*	jun B proto-oncogene	2.01	8.34	Transcription factor
*MONDOA*	Mlx interactor		2.26	Transcription factor
*SOX4*	SOX4 gene for SRY (sex determining region Y)-box 4		1.97	Transcription factor
*BHLHB2*	Basic helix-loop-helix domain containing, class B, 2	4.26 (2)	4.61 (2)	Transcription factor
*SNAI2*	Snail homolog 2 (*Drosophila*)		2.37	Transcription factor

^a,b^The primary sequence names and descriptions for the differentially regulated genes (defined as greater or equal to 1.7-fold changed with a p-value of < 0.01) were extracted using Rosetta Resolver. Genes from the dominant ontology classes are shown and thus genes with unknown function or those not in well represented ontology classes are not listed. The complete set of differentially expressed genes can be found in Additional files 6, 7, 8, 9. ^c^Fold change in expression of the induced or repressed genes in the MDA-MB-231 CN cell line after a 24 h exposure to 2 ng/ml recombinant TGF-β1 compared to the diluent treated controls. The number in parentheses after the fold change indicates the number of affected elements that were averaged to calculate the fold difference. ^d^Fold change in expression of the induced or repressed genes in the MDA-MB-231 H2 cell line after a 24 h exposure to 2 ng/ml recombinant TGF-β1 compared to the diluent treated controls. The number in parentheses to the right of the fold change indicates the number of affected elements that were averaged to calculate the fold difference. ^e^The gene ontology annotation was curated from the Summary Function and GO fields downloaded from SOURCE [105].
