# Supplementary material for: HER-2 overexpression differentially alters transforming growth factor-β responses in luminal versus mesenchymal human breast cancer cells
Source: Breast Cancer Res. 2005 Nov 8;7(6):R1058–79. doi: 10.1186/bcr1343 (PMC1410754; doi:10.1186/bcr1343)
Supplement: Additional File 10 — Table (Excel file) of the expression differences in MCF-7 CN versus MCF-7 H2 cells of genes in, or that modulate, the TGF-β signaling pathway. Genes listed in red are higher in MCF-7 H2 relative to MCF-7 CN and those listed in green are lower in MCF-7 H2 relative to MCF-7 CN. The first four columns are from one experiment and the second four columns are from an independent experiment using different treated flasks of cells. [file bcr1343-S10.pdf]

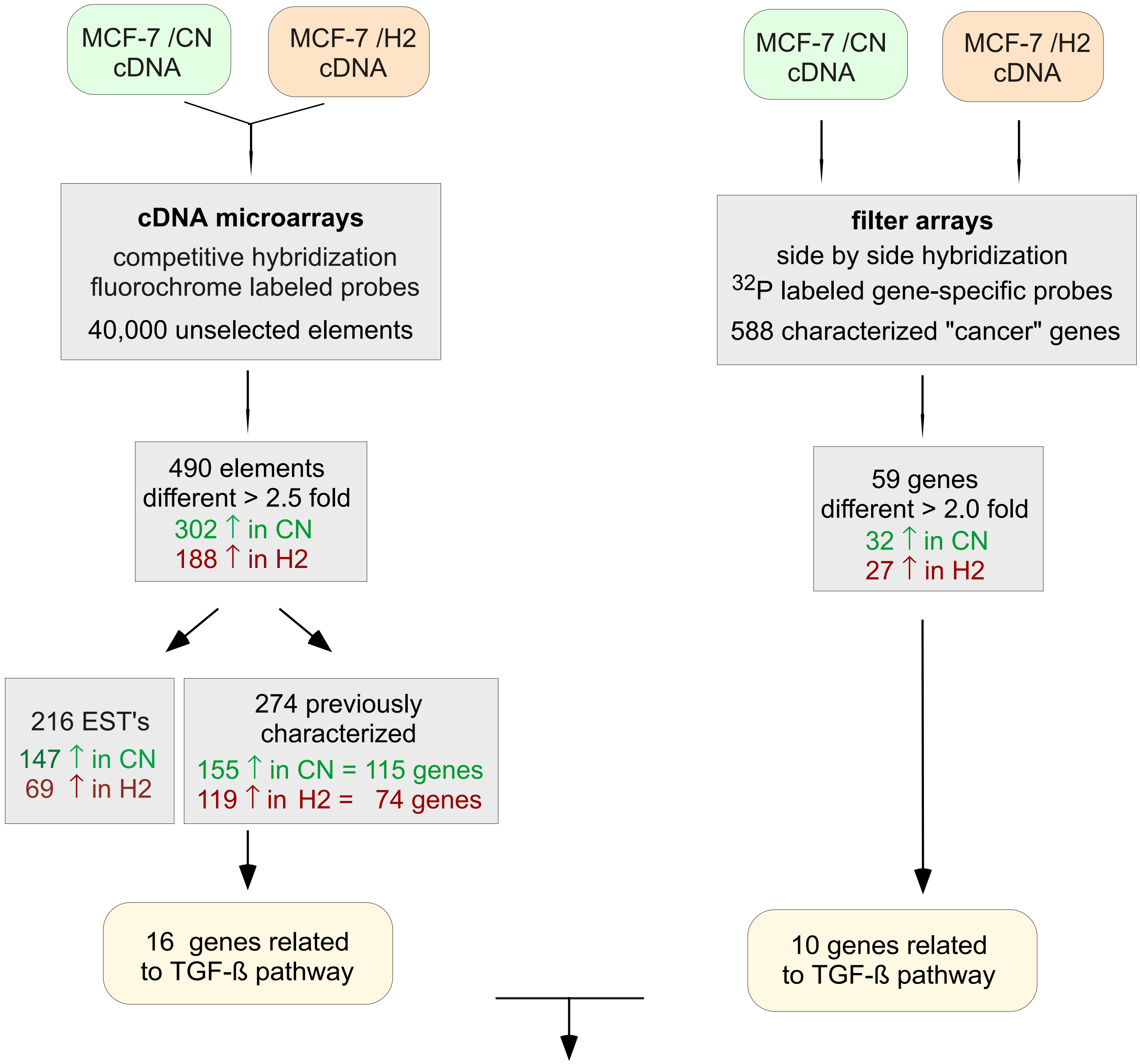

**Ligands**	cDNA	filter
TGF-ß2 (M19154)	2.5	2.0
TGF-ß3 (J03241)	-	2.8
BMP-3 (M22491)	-	2.8
BMP-5 (M60314)	7.1	4.5
BMP-7 (M60316)	2.5	3.9
**Receptors**		
TBR II (D50683)	2.5	-
Endoglin (X72012)	2.9	-

**TGF-ß Targets**	cDNA	filter
CTGF (M92934)	5.2^{*3}	4.4
Cyr61 (AF031385)	3.8^{*2}	-
B-Ig-H3 (M77349)	2.6	-
Endothelin1(J05008)	2.7	-
Timp-2 (J05593)	2.9	2.0
Col3A1(X14420)	2.9^{*2}	4.4
Col5A1(M76729)	4.0^{*3}	-
Col18A1(AF018081)	3.7	4.7
IGFBP5 (R36012)	4.1^{*3}	2.2
CD44 (X55150)	2.8^{*2}	2.1
IGFR1(X04434)	3.1	2.4
